# Pesticide Application Practices and Knowledge among Small-Scale Local Rice Growers and Communities in Rwanda: A Cross-Sectional Study

**DOI:** 10.3390/ijerph16234770

**Published:** 2019-11-28

**Authors:** Benjamin Ndayambaje, Hellen Amuguni, Jeanne Coffin-Schmitt, Nancy Sibo, Martin Ntawubizi, Elizabeth VanWormer

**Affiliations:** 1 Global Health Delivery, University of Global Health Equity, 6955 Kigali, Rwanda; nancynana68@gmail.com; 2 School of Natural Resources, University of Nebraska-Lincoln, Lincoln, NE 68583, USA; liz.vanwormer@unl.edu; 3 Infectious Disease and Global Health, Tufts Cummings School of Veterinary Medicine, North Grafton, Massachusetts, MA 01536, USA; janetrix.amuguni@tufts.edu (H.A.); jlc558@cornell.edu (J.C.-S.); 4 School of Veterinary Medicine, University of Rwanda, 57 Nyagatare, Rwanda; martin.ntawubizi@gmail.com; 5 School of Veterinary Medicine and Biomedical Sciences, University of Nebraska-Lincoln, Lincoln, NE 68583, USA

**Keywords:** agricultural runoff, sustainable agriculture, Rwanda, pesticides, farmer knowledge, ecosystem health

## Abstract

Background: Agriculture contributes a third of Rwanda’s GDP and is the main source of income for rural households, with 80% of the total population involved in crop and/or livestock production. The Government of Rwanda established the Muvumba rice project in 2011 amidst a policy shift towards rice as a national staple crop. However, the indiscriminate use of pesticides by local, low-income rice growers has raised concerns about potential human, animal and ecosystem health impacts as pesticide distribution and application are not strictly regulated. Although pesticide use can directly influence farmer health and ecosystems, little is known about small-scale farmers’ pesticide application practices and knowledge. We aimed to assess local application practices and understanding of pesticides to identify gaps in farmers’ knowledge on safe pesticide use and deviations from established standards and recommended practices. Methods: We conducted a cross-sectional study consisting of observations of pesticide practices and interviews with 206 small-scale rice growers in Nyagatare District, Rwanda, in March 2017. Descriptive statistical analyses (sample means, standard deviation and range) were performed, and we evaluated the association between farmers’ personal protective equipment (PPE) use and their education level and literacy status. Results: Over 95% of observed farmers did not comply with minimum standards for safe pesticide use, and 80% of respondents reported that they stored pesticides in their homes without personal protection measures. Education and literacy level were not significantly associated with PPE use. Additionally, 90% of respondents had experienced adverse health effects after using pesticides including intense headache, dizziness, stomach cramps, skin pain and itching, and respiratory distress. All respondents also reported animals in and around the rice scheme (cattle, birds, and fish) behaving abnormally or with signs consistent with pesticide exposure in the six months preceding the study, which may be linked to pesticide-contaminated water. Conclusions: Our study demonstrates potential for high exposure to pesticides for farmers, their families, and animals sharing rice-growing or downstream environments and points to the need for training on safe and effective pesticide use.

## 1. Introduction

Agriculture plays a vital role in sustainable development and poverty reduction, especially in many low- and middle-income countries [1]. However, agricultural yield commonly faces losses due to pests—organisms that cause plant disease and decreased growth [2]. Different technologies have been developed to protect diverse crops from pests, but pesticide application is one of the most common practices globally [3]. Agricultural pesticides include diverse chemicals commonly used by farmers to repel, destroy or control the impact of pests [3]. While these chemicals can be very effective at reducing pest damage, they also have the potential to adversely impact human, animal, and ecosystem health [4,5].

In Rwanda, agriculture provides the main source of income for rural populations and contributes one third of the country’s gross domestic product [6]. Rice (*Oryza* spp.) is being promoted by the government as one of the main staple crops across Rwanda, and the Muvumba rice scheme was created in 2011 as an alternative to ancestral livestock-keeping activities in Nyagatare District. Approximately 700 hectares were reserved for Indian rice production investors (Nyagatare Agrovenger Rwanda [NAVR] Ltd) and 1000 hectares were distributed among individual Rwandan farmers and a community cooperative named Cooperative des Producteurs de Riz de Muvumba (COPRIMU) [7]. In Rwanda, rice is grown twice yearly in two agricultural seasons: season a, which spans September to February, and season b, which starts in March and ends in July of the same calendar year [8]. Rwanda has intermittent rain patterns with a short rainy season from September to November and a longer, heavier rainy season between March and May. A short dry period occurs from December to February, and a longer one lasts from June to August. Rainfall ranges from about 900 mm in the east and southeast to 1500 mm in the north and northwest volcanic highland areas [9].

Rwandan rice farmers within and beyond the Muvumba Valley rely heavily upon the use of pesticides to prevent pest-related yield losses. Muvumba River water is typically used to mix pesticide (powder or liquid form) in a bucket. Farmers then use backpack sprayers filled with mixed water and pesticide to spray in the rice fields. In field interviews, local farmers reported commonly using different types of pesticides including herbicides (propanil, butachlor, and pendimenthalin), fungicides (carbendazim and propiconazole), and insecticides [organophosphates (Chlorpyriphos), pyrethroids (cypermethrin, profenofos, and a mixture of these two [Roket]), and neonicotinoids (imidacloprid)]. Butachlor and carbendazim are classified as unlikely to cause acute hazard in normal use (Class U), and propinil and pendimenthalin fall into a moderately hazardous class (Class III) [10]. However, propiconazole and the insecticides listed above are all classified as moderately hazardous (Class II) by the World Health Organization [10]. These pesticide groups can cause acute toxicity in mammals following oral administration, with low toxicity if inhaled or applied to skin. Moreover, some of these pesticides such as imidocloprid are highly toxic to birds by acute oral administration [11]. Persistence of these pesticides varies based on the particular product and local environmental characteristics such as soil type, but some of the pesticides used by local farmers can persist in soil for months, or even years [12,13,14]. Although these pesticides are commonly used by rice growers, the knowledge, practices and agricultural factors influencing how local farmers apply pesticides and the differences between local application and international recommended safe practices are unknown. We hypothesized that low knowledge of pesticide toxicity and poor application practices present a health risk to local farmers, communities, animals, and ecosystems. We also hypothesized that the education and literacy levels of farmers are associated with safe pesticide practices such as use of personal protective equipment (PPE). To assess farmer knowledge and application of pesticides, we interviewed small-scale rice growers and observed local pesticide application approaches including mixing, storage, and use of personal protective equipment (PPE). This information is crucial in order to enhance safe pesticide use to protect farmers, animals, and the environment from pesticide-related health impacts in Rwanda.

## 2. Materials and Methods 

### 2.1. Target Areas and Population

**Figure 1:**
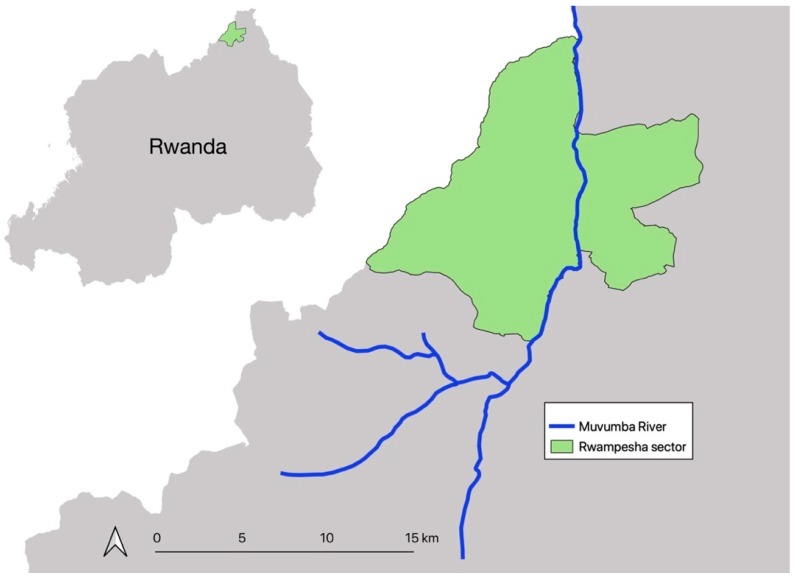
Map of the Muvumba River study area in Rwempasha sector in northeastern Rwanda.

In March, 2017, we conducted a cross-sectional study consisting of observations of pesticide practices and interviews with farmers involved in rice production in Eastern Rwanda. Using a random sampling approach, we selected 206 study participants from 600 rice farmers in Rwempasha sector along the Muvumba Valley marshland (Figure 1). The site was selected based on the crop grown (rice), reports of pesticide usage, cooperation from local rice producers and rice-growing cooperative leaders, and willingness of farmers to participate.

The Muvumba Valley marshland is located in Nyagatare District in the Eastern Province of Rwanda (20°35′56.8″ S, 29°43′46.6″ E; 1767 m of altitude) [8]. Rice production started in 2010 in upstream areas and progressively covered the lower parts of the marshland. In addition, canals that irrigate the rice are channeled from the Muvumba River to dams and secondary channels to feed the rice cultivation plots [8].

### 2.2. Data Collection and Analysis

Trained enumerators conducted interviews in Kinyarwanda with rice farmers working in the Muvumba Valley marshland. The study participants were 18 years of age or older, with at least one year of experience working in a rice scheme. Farmers working outside of the study area and those who were unable to give informed consent were not included in the study. A pre-study consultation meeting was organized with different stakeholders involved in the Muvumba rice farming project to discuss local collaboration and possible benefits of the research.

The study participants were observed using pesticides for five days before being engaged for interviews. We gathered information on farmer pesticide application practices using an observation sheet, which included eight international standards of good pesticide practices. Farmer behaviors observed included mixing pesticides (powder and liquid) before spraying, cleaning materials after spray/use, storage and disposal of empty containers, and use of personal protective equipment (PPE). The observation sheet was developed from a literature review and international guidelines on the proper use of pesticides [15]. The sheet focused on the proper calibration of spraying equipment, whether the farmer was aware of exact field location to be treated, timing of pesticide spraying, the presence of buffer zones between the sprayed area and a water source (15–30 m recommended), and proper storage facilities.

Following the observation session, a structured questionnaire on farmer knowledge and perception of pesticide use, handling, and application was administered based on published literature and local context. We divided the questionnaire into three sections: (1) social and demographic information about the subjects, (2) participant knowledge about pesticides and their application, and (3) participant knowledge of the potential impacts of pesticide use on human, animal and environmental health. Questionnaires were tested for clarity, completeness, and participant understanding with 10 rice farmers in a sector outside of the study area.

Questionnaires, observation sheets, and interview guides were approved by the University of Global Health Equity Institutional Review Board (Approval ID: 0001). Permission was granted by district authorities and cooperative leadership to conduct the surveys and observational visits in the Muvumba rice scheme. The study participants were given informational handouts about the proper use, handling, and health effects associated with pesticides following the interviews. Following translation from Kinyarwanda to English, field questionnaire and observation data were entered into Microsoft Excel for data management and cleaning. Descriptive statistical analyses (calculation of sample mean, standard deviation (SD) and range) were performed using STATA software (StataCorp, College Station, TX, USA). Chi-square tests were used to evaluate factors (education level, age, farming site, farming experience and literacy) associated with good pesticide practices. A significance level of *α* = 0.05 was used for all statistical tests.

### 2.3. Ethics Approval and Consent to Participate

This study was reviewed by University of Global Health Equity—IRB #0001, and all study participants consented before the interviews.

## 3. Results

### 3.1. Personal Protective Equipment (PPE) Use and Pesticide Mixing and Storage

Observed and self-reported pesticide practices showed limited farmer use of recommended methods for safe spraying, mixing and storage (Table 1 and Table 2). All study participants were observed spraying pesticides without wearing shoes or protective boots, with almost everyone wearing their normal clothing (99.5%; 205 of 206 farmers). Less than 11% of the farmers observed used any PPE. Of those who used PPE, glasses/goggles were the most commonly used (6.8%). The majority of study participants (92.7%; 191 farmers) were observed using their bare hands to mix the powder pesticides with water before spraying. A higher percentage of study participants (23%; 47 farmers) reported using complete PPE during interviews than were observed using complete PPE in the field (Table 2). A complete set of PPE included goggles for eyes, a respirator, gloves, work boots or rubber “gum” boots, and a plastic apron to protect the farmers from pesticide contact. The majority of interviewed participants (77%) reported using incomplete PPE as they were missing at least one of these protective elements. Although age, gender and educational level differed among the study participants (Table 2), PPE use was similar among the categories for each of these variables. None of the demographic or farming factors tested were significantly associated with PPE use (*p*-values > 0.05; Table 2).

During household observational visits, we observed that all study participants stored pesticide in powder or liquid form in their homes. Similarly, all study participants reported storing pesticides or empty pesticide cans in their premises (house/kitchen). The empty cans were used for fetching water, serving water to calves, and as containers for drinking water for children.

**Figure 2:**
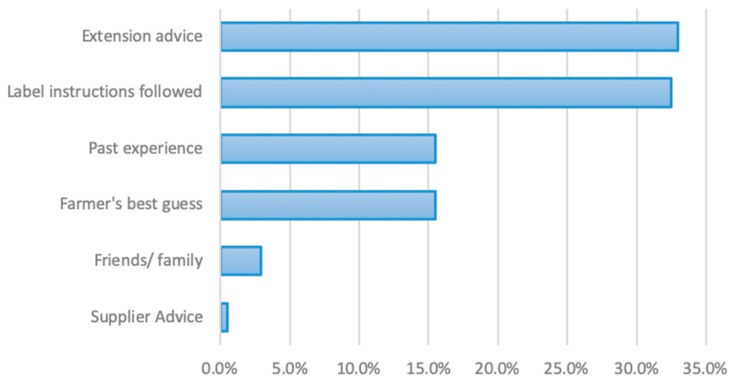
Reported sources of information on mixing pesticides used by small-scale rice farmers.

Farmers relied upon different primary sources of information about safe pesticide mixing, storage, and application (Figure 2). The majority of farmers relied upon available extension services (33.0%; 68 farmers) or pesticide label information (32.5%; 67 farmers) when seeking information about appropriate mixing techniques. However, many farmers relied upon either personal experience (15%; 32 farmers) or their best guess (15%; 32 farmers) when mixing pesticides.

### 3.2. Compliance with International Good Pesticide Practice Standards

Overall, we observed poor compliance with the international standards of good pesticide practices established by FAO and the WHO [15,16] across the study participants (Table 3). These standards included maintaining working equipment in good condition, displaying post spraying signs, using application techniques that increase efficiency and allow the lowest effective labeled application rate, avoiding unnecessary and poorly timed application of pesticides, avoiding overspray and drift, timing pesticide application appropriately and reducing the potential for off-site transport, establish buffering zones, and avoiding repetitive use of the same pesticide.

A greater proportion of the farmers (20%; 41 farmers) was compliant with the 6th standard (timing pesticide application in relation to soil moisture and anticipated weather conditions, and reducing the potential for off-site transport), followed by standard 4 (avoiding unnecessary and poorly timed application of pesticides) with (16%; 32 farmers). The 7th standard (establishing buffer zones where pesticide is not applied a safe distance (minimum of 20 to 30 meters recommended) from wells and surface water) was the least practiced (2.9%; 6 farmers). Only nine study participants (4.4%) were compliant with at least half of the standards of good practice.

All of the study participants reported that they sprayed pesticides more than twice (the recommended spaying frequency) per growing cycle. The study participants attributed the need for additional spraying to the ineffectiveness of the pesticides, increased resistance of pests towards the pesticides, and abundance of diseases. Moreover, some farmers (78%; 161 farmers) also reported experimentally increasing the pesticide dosage during the mixing stage in order to observe the effect of pesticides used on the control of pests and diseases.

### 3.3. Farmers’ Knowledge of Pesticide Use and Perceived Health Impacts of Pesticides

The majority of the study participants (99%; 204 farmers) believe PPE and regular pesticide safety trainings can protect them from adverse health effects of pesticides. However, only 28% (58 farmers) had previously received any information on pesticide use from various agriculture-related stakeholders (government, NGOs, or peers). Farmers participated in general pesticide training from the Rwanda Agricultural Board (RAB; 23.3%; 46 farmers), sector agronomists (16%; 33 farmers), or non-governmental organizations (2.4%; five farmers). Only four farmers (1.9%) received specific training on human health and safety of pesticides, including safe disposal of pesticides. No trainings were delivered on effects of pesticides on animal and environmental health or on strategies for reducing pesticide use such as Integrated Pest Management (IPM).

All study participants reported adverse health effects during or after pesticide spraying in the field. Farmers reported experiencing one or more of the following symptoms: itchy skin, headaches, difficulty breathing, and nausea or stomach upset. Only three farmers (1.5%) could explain the connections between pesticide use and negative impacts on aquatic life and the environment. However, all participants directly observed or had heard reports of cows, birds or fish showing signs of pesticide intoxication (such as death, uncoordinated movement, and weakness) within 30 minutes to six hours after consumption of pesticide or presence in recently sprayed areas. Almost half of study participants (48.5%; 100 farmers) also reported seeing small fish die a few minutes after applying the pesticides, especially during plot preparations before planting rice.

## 4. Discussion

Poor pesticide handling, application, and storage can negatively impact the health of humans, animals, and ecosystems [17]. Our study on pesticide knowledge and practices among small-scale local rice growers provides information on existing health risks and actions that can be taken to protect the health of farmers, domestic animals, wildlife, and their shared environments in and beyond Rwanda.

The limited use of personal protective equipment (PPE) reported and observed among a majority of small-scale rice farmers likely poses a risk to individual and family health. Although few participants in our study were formally trained on health impacts of pesticides, all participants recognized acute changes in their health after starting to use pesticides including skin, eyes and nasal passage irritations, headache and stomach cramps. Some farmers may therefore understand health risks but ignore PPE recommendations due to practical and financial challenges. Farmers participating in our study commonly reported that they did not follow PPE recommendations because (1) the recommended rubber boots (also called gumboots) frequently get stuck in mud when moving across the field during spraying, and (2) they had no financial capacity to purchase PPE. Similarly, low levels of PPE usage were reported in Uganda and Kenya [18,19], where the majority of farmers did not use PPE due to poor knowledge about the adverse impact of pesticides on human health as well as financial constraints. In West Africa (Ghana), where the majority of farmers studied were aware of the negative impacts of pesticides on health, 65% did not use full PPE when handling pesticides [20]. Therefore, interventions targeting financial and other factors in addition to farmer knowledge may be required to improve PPE use and safe pesticide handling.

Our study revealed no significant association of education (formal education level, farming experience, or literacy) with PPE use. In contrast, links between increased education and safe pesticide practices have been observed in other low and middle-income countries. In Egypt, farmers with school education used gloves more frequently than those with no school education, but no significant differences were observed in the use of protective clothing or masks [21]. The use of masks and gloves was more common in farmers with higher education levels in Mexico, but education level did not influence other pesticide practices such as washing equipment or storing pesticides in the home [22]. This suggests that the impact of formal education varies across types of pesticide practices and geographic areas. For the surveyed farmers in Rwanda, the absence of a significant association between education and PPE use may be due to lack of coverage of safe pesticide use or broader agricultural safety topics in the formal primary and ordinary (through 9th grade level) education curriculum. In addition, language barriers may complicate a farmer’s ability to understand pesticide labels regardless of literacy status. Many labels and application manuals are written in foreign languages such as English and Chinese, limiting the ability of farmers who can read local language text to understand this information. Inability to interpret written safety instructions due to low levels of literacy and labels being written in English has also been reported in rice farmers in Tanzania [23]. While increasing formal education levels of farmers may enhance their ability to read labels in local languages or English, targeted pesticide safety trainings may be more effective for enhancing safe pesticide practices to protect human, animal, and ecosystem health than formal schooling.

Improper pesticide storage practices may be an additional important source of exposure for humans and animals in the study area. All study participants stored full, partially full, or empty cans of pesticides in their kitchen or other areas of their homes and were observed using empty pesticide cans for fetching water for domestic purposes such as washing clothes, cooking and drinking. The proportion of surveyed farmers storing pesticides at home was higher than those reported in Tanzania (68%) [24] and Ethiopia (43%) [25], which suggests a critical local need for trainings on safe pesticide storage and management.

The Muvumba River serves as an important water source for irrigation and domestic purposes (drinking, cooking, washing clothes, watering home gardens) as well as for local domestic and wild animal populations. The reported pesticide use behaviors pose potential health risks to the communities in and around the Muvumba River as well as communities downstream, which may be exposed to water contaminated by pesticide runoff. Human environmental contact with pesticides is most likely to occur through occupational exposure [26] but can also occur through exposure to contaminated water sources [27,28,29]. The workers who mix, transport, load, and apply the pesticides are at high health risk. However, even the family members who use old pesticide containers for food or water storage and people drinking at downstream water sources are at risk of pesticide intoxication [30,31,32]. Widespread exposure to pesticides such as neurotoxic organophosphates has resulted in extensive poisonings and deaths, especially in developing countries, and may additionally put children at risk for neurodevelopmental disorders and cognitive deficits [33,34]. Moreover, pesticides can be toxic to non-targeted organisms including fish, birds, beneficial soil microorganisms and insects, and other wildlife [35,36]. In addition to acute toxicity, sub-lethal effects of widely used neonicotinoid insecticides have reduced wild bee populations and threaten bee biodiversity [37]. Although the human symptoms and observed animal clinical signs or death reported by farmers in this study related to acute pesticide exposure, long-term pesticide exposure may also lead to chronic health issues [38,39,40,41].

This cross-sectional study was based on field observations of farmer practices and self-reported information. There are chances of observer (enumerator) and recall (by farmers) bias. However, the good relationships among the University of Rwanda (UR) and University of Global Health Equity (UGHE) with the study communities, the sample size of over 200 farmers, and our ability to compare observations with self-reported information suggest that our findings on risky pesticide management practices reflect the behavior of the majority of rice farmers in the Muvumba River area. Given the health risks associated with current pesticide practices, there’s an urgent need for regular trainings on pesticide handling, application and management, practical approaches to using PPE in local conditions (e.g., muddy fields), and strategies such as Integrated Pest Management (IPM) to decrease pesticide use among rice farmers in Rwanda. Increased PPE accessibility and availability at agricultural shops and affordable prices for farmers are needed. In addition, laws and regulations of pesticide selling, application and monitoring the use should be reinforced by relevant authorities.

## 5. Conclusions

Our study suggests high potential for exposure to pesticides due to limited basic pesticide safety practices and potentially dangerous health shortfalls in and around the Muvumba Valley rice scheme of Nyagatare District, Rwanda. Specifically, the knowledge and skills of farmers appeared to be inadequate to prevent potential hazards and risks (water contamination; killing of birds, fish, beneficial insects, or non-target plants) associated with the use and management of pesticides. Urgent comprehensive intervention measures are needed to reduce major health risks to small-scale rice farmers, their families, and surrounding animals and ecosystems. Such measures could include access to personal protective equipment (PPE), pesticide application and storage training before use, pesticide and health inclusion in the formal education curriculum, adequate warning descriptions in local languages, and measures to reduce cost barriers to the adoption of safe behaviors.

## Figures and Tables

**Table 1:** Observed pesticide handling practices used by small-scale rice farmers (*n* = 206) in Nyagatare District, Rwanda.

Activity	Pesticide Handling Practice	Number of Farmers (Percentage)
Use of personal protective equipment during pesticide spraying	No footwear worn (bare feet) (dangerous)	206 (100%)
	Normal clothes worn (dangerous)	205 (99.5%)
	No gloves worn (bare hands) (dangerous)	191 (92.7%)
	Boots owned, but not worn (dangerous)	22 (10.7%)
	Glasses/goggles (safe)	14 (6.8%)
	Gloves worn (safe)	13 (6.3%)
	Plastic apron worn (safe)	0 (0%)
	Respirator worn (safe)	0 (0%)
Pesticide mixing method	From a can/plastic tub (safe)	14 (6.8%)
	Gloves worn (safe)	13 (6.3%)
	Powder mixer stick used (safe)	0 (0%)
Pesticide storage	In the house (dangerous)	206 (100.0%)
	In the kitchen (dangerous)	23 (11.2%)

**Table 2:** Factors associated with self-reported personal protective equipment (PPE) use by small-scale rice farmers surveyed in Nyagatare District, Rwanda.

Socio-Demographic Factors		Total Farmers in Each Category (*n* = 206) and Percentage (%)		Incomplete PPE			Complete PPE	*p*-Value
				(*n* = 159, 77%)			(*n* = 47, 23%)	
		*n*	%	*n*	%	*n*	%	
Education level	None	36	17.5	28	77.8	8	22.2	
	Elementary/Primary (6th grade level)	147	71.4	114	77.6	33	22.5	
	Ordinary (9th grade)	23	11.2	17	73.9	6	26.1	0.924
Age	≤19	2	0.97	2	100	0	0	
	20–42	152	73.8	118	77.6	34	22.4	
	43+	52	25.2	39	75	13	25	0.688
Gender	Female	38	18.5	26	68.42	12	31.58	
	Male	168	81.6	133	79.2	35	20.8	0.154
Years of experience growing rice	≤1	81	39.3	61	75.3	20	24.7	
	2	24	11.7	21	87.5	3	12.5	
	3	45	21.8	35	77.8	10	22.2	
	4+	56	27.2	42	75	14	25	0.621
Literacy	Literate	156	75.7	119	76.3	37	23.7	
	Illiterate	50	24.3	40	80	10	20	0.586
Farm type	Commercial (Company)	100	48.5	74	74	26	26	
	Cooperative	82	39.8	69	84.2	13	15.9	
	Individual	24	11.7	16	66.7	8	33.33	0.114

**Table 3:** Observation of rice farmers’ compliance with established standards of good pesticide practice.

Established Standard	Number (Percent) of Farmers Compliant
Standard 1 (Maintaining the working equipment in good condition)	23 (11.2%)
Standard 2 (Presence of post spraying signs)	31 (15.1%)
Standard 3 (The application techniques that increase efficiency)	18 (8.7%)
Standard 4 (Avoid unnecessary and poorly timed application of pesticides)	32 (15.5%)
Standard 5 (Avoid overspray and drift especially near surface water body)	23 (11.2%)
Standard 6 (Time pesticide application and reduce the potential for off-site transport)	41 (19.9%)
Standard 7 (Establish buffer zones minimum 50–100 meters recommended from well)	6 (2.9%)
Standard 8 (Avoid repetitive use of the same pesticide)	22 (10.7%)
Compliant with ≥4 standards	9 (4.4%)
Compliant with <4 standards	197 (95.6%)

## Abbreviations

PPE	Personal Protective Equipment
FAO	Food and Agriculture Organization
WHO	World Health Organization
NGO	Non-Governmental Organization
